# Remating and Sperm Competition in Replicate Populations of *Drosophila melanogaster* Adapted to Alternative Environments

**DOI:** 10.1371/journal.pone.0090207

**Published:** 2014-02-25

**Authors:** Devin Arbuthnott, Aneil F. Agrawal, Howard D. Rundle

**Affiliations:** 1 Department of Biology, University of Ottawa, Ottawa, Ontario, Canada; 2 Department of Ecology and Evolutionary Biology, University of Toronto, Toronto, Ontario, Canada; North Carolina State University, United States of America

## Abstract

The prevalence of sexual conflict in nature, as well as the supposedly arbitrary direction of the resulting coevolutionary trajectories, suggests that it may be an important driver of phenotypic divergence even in a constant environment. However, natural selection has long been central to the operation of sexual conflict within populations and may therefore constrain or otherwise direct divergence among populations. Ecological context may therefore matter with respect to the diversification of traits involved in sexual conflict, and if natural selection is sufficiently strong, such traits may evolve in correlation with environment, generating a pattern of ecologically-dependent parallel evolution. In this study we assess among-population divergence both within and between environments for several traits involved in sexual conflict. Using eight replicate populations of *Drosophila melanogaster* from a long-term evolution experiment, we measured remating rates and subsequent offspring production of females when housed with two separate males in sequence. We found no evidence of any variation in male reproductive traits (offense or defense). However, the propensity of females to remate diverged significantly among the eight populations with no evidence of any environmental effect, consistent with sexual conflict promoting diversification even in the absence of ecological differences. On the other hand, females adapted to one environment (ethanol) tended to produce a higher proportion of offspring sired by their first mate as compared to those adapted to the other (cadmium) environment, suggesting ecologically-based divergence of this conflict phenotype. Because we find evidence for both stochastic population divergence operating outside of an ecological context and environment-dependent divergence of traits under sexual conflict, the interaction of these two processes is an important topic for future work.

## Introduction

Sexual conflict occurs when the reproductive interests of the two sexes are not aligned, leading to sex-specific selection on shared traits [1]. When the trait is controlled by different loci in males and females (e.g., mating rate), sexually antagonistic selection can favor alleles that increase a male's reproductive success relative to other males, even if this comes at a cost to female fitness. Similarly, selection can also favor alleles in females that increase their resistance to such male-induced harm, even if this reduces male fitness [2]. Such interlocus sexual conflict can drive a process of ongoing antagonistic coevolution in which changes in one sex generate renewed selection on the other [3], [4]. The economics of the resulting conflict in terms of sex-specific costs and benefits can have large impacts on the fitness of males and females [3], [5]–[7], and by extension, population mean fitness [8]–[10].

Interlocus sexual conflict is common in nature and has led to a great diversity of traits that serve to increase the reproductive success of males at the expense of females (e.g., persistent male courtship and harassment, toxic ejaculates, spiny genitalia), as well as traits that provide resistance in females (e.g., escape behavior, complicated reproductive tracts; [2]). The particular traits that evolve in any given case are thought to be the product of chance events such as the order in which mutations occur or the standing genetic variation in a population. Because of the persistent and potentially strong selection sexual conflict can generate, traits involved in sexual conflict may diverge rapidly among populations [11], [12] Consistent with this, cases of remarkable diversity in conflict-mediating traits have been documented even among recently isolated populations and species [13]–[15]. In addition to generating phenotypic diversity, such divergence may contribute to the evolution of reproductive isolation [16]–[18], leading to the suggestion that sexual conflict may be an important engine of speciation [19], [20]. Given the central role of sexual conflict in the evolution of mating interactions, sex-specific and population mean fitness, and population divergence/speciation, understanding how traits involved in sexual conflict diverge among populations is an important goal in evolutionary biology.

Divergence through sexual conflict is driven by sexually antagonistic selection and can therefore occur even in the absence of ecological differences between populations. This has led some to classify sexual conflict as a non-ecological promoter of speciation [21], [22]. However, the traits involved in sexual conflict may also affect non-sexual fitness and may thus be subject to natural selection within an ecological context, and ecology may alter the opportunity and nature of sexual conflict [1], [23]–[26]. While natural selection and the environment have been shown to interact with sexual conflict and mating system evolution within populations [23], only recently has this been extended to studies of sexual conflict's role in population divergence/speciation.

An emerging appreciation of the importance of ecology is suggested by several empirical studies of sexual conflict that included different environments, either through an experimental manipulation or by exploiting naturally occurring variation. For example, experimental populations of seed beetles adapting to a new food showed less diversification of traits involved in sexual conflict relative to populations continuing to evolve on the same food as their ancestor [27]. In a separate experiment also in seed beetles, populations maintained under conditions in which the duration of the reproductive period was altered were shown to have evolved differences in some genital and mating traits [28], [29]. In freshwater isopods, male harassment of conspecific females has been shown to differ between habitats differing in predation, demonstrating ecologically-based differences (and divergence) in the opportunity for sexual conflict [30]. Similarly, the strength of predation has been shown to influence the patterns of genital divergence in mosquitofish [31]. Nevertheless, our understanding of the impact of ecological differences on traits involved in sexual conflict is limited.

In a previous test of ecology's influence on the divergence of traits involved in sexual conflict, we demonstrated ecologically-dependent parallel evolution of male harm and female resistance among replicate populations of *Drosophila melanogaster* that had independently adapted to two different food environments containing either ethanol or cadmium [32]. Specifically, we showed that males from populations that had independently adapted to ethanol decreased female longevity more than did males from replicate populations independently adapted to cadmium. Ethanol-adapted females from these populations were also more resistant to male-induced reductions in longevity, on average, as compared to females from the replicate cadmium-adapted populations. Such ecologically-dependent parallel evolution of a key life history trait demonstrates that ecology can play a central role in shaping traits involved in sexual conflict.

Here we extend work on these populations by assaying divergence in female remating rate and male reproductive offense and defense. These are traits that are commonly thought to evolve by sexual selection in general, with a strong role for sexual conflict in particular. This contrasts with our past study on longevity, a trait on which natural selection is also likely to be strong. Of particular interest is the relative importance of among-population divergence within environments as compared to ecologically-mediated divergence between environments (i.e. parallel evolution in correlation with environment). We find some evidence for divergence among populations both within and between environments in females (but not in males), suggesting roles for both environment-independent sexual conflict and ecologically-divergent natural selection in the evolution of these traits.

## Materials and Methods

### Experimental populations

For a detailed description of population origin and maintenance, see [32]. In brief, in 2007 a laboratory-adapted *Drosophila melanogaster* stock was split into 20 independent, isolated populations, with ten of these maintained on food supplemented with 12% ethanol and the other ten on food containing 70 ug/mL cadmium chloride. Like the stock, which continued to be maintained on standard (i.e. lacking ethanol and cadmium) food, these experimental populations were maintained in population cages with overlapping generations. After four years, a reciprocal transplant assay measuring the number of adult offspring produced by replicate male-female pairs demonstrated that each of the 20 experimental populations had increased in fitness relative to the stock when tested in its evolved environment. Although adaptation to cadmium also conferred increased fitness in ethanol as a by-product (the converse did not occur), each population also produced significantly more offspring on average in its evolved environment than in the other selective environment, demonstrating the adaptive divergence of these populations between environments [32]. For subsequent measures of reproductive traits, we selected four populations from each environment, chosen as those with the highest fitness in their evolved environment as these populations showed the greatest response to the imposed environmental selection. These are the same populations as used in [32].

In each assay below, we collected flies from each of the eight experimental populations, the stock population, and a population fixed for a dominant visible mutation (brown eyes: *bw* ^D^). All flies were reared for a single generation in standard (i.e. ancestral) stock food in vials with 50 or fewer individuals. Shortly after initial exposure to the experimental environments, development time was greater among the ethanol populations (in the ethanol environment). However, development time was similar among all populations in the standard environment at the time of this study. Virgins were collected on days 10–11 of their life-cycle within 8 h of emergence using light CO_2_ anesthesia, and sexes were held separately (10 females/vial or 7 males per vial) for 3 d prior to conducting an assay. All three assays employed a design similar to that used for measuring sperm offense and defense in *Drosophila* in that a single female was held individually with two different males in sequence. One of the two males was from the *bw* ^D^ stock whereas the other was not, allowing mating with each male to be inferred by presence of any offspring of the matching phenotype (i.e. mutant vs. wild-type). Unlike a standard sperm competition assay, the female was held in the same vial throughout, ensuring that offspring from the first male would be present (provided she mated with this male) and allowing the timing of mating, as well as a male's sperm competitive ability, to contribute to the relative frequency of their offspring. The three assays separately varied the identity of the female (assay one: female remating), the first male (assay two: male reproductive defense), or the second male (assay 3: male reproductive offense), as described below.

### Female remating and offspring production

This assay tested whether females from the different populations and environments varied in their propensity to remate, and also quantified their subsequent offspring production using sperm of the two males. In each trial, a single female from one of the experimental populations (i.e. cadmium or ethanol-adapted) was placed in a vial of standard food along with a single wild-type stock male and left for 6 h. We previously found that 6 h was sufficient to ensure the vast majority of females (98%) had mated (D. Arbuthnott, unpublished data). It is possible that some females mated more than once during this period, thereby influencing patterns of subsequent remating and offspring production. However, such variation in multiple mating frequency arising from either sex is relevant to reproductive defense and offense and could potentially underlie any detected variation in this.

After this initial mating period, the stock male was removed and immediately replaced with a single *bw* ^D^ male. These marker males were given the opportunity to mate the experimental female for 8 h, after which they were removed. Females were allowed to lay eggs in the same vial for an additional 17 h, after which they were discarded. Because the allelic marker in the second male is dominant, offspring sired by that male will express the brown-eye phenotype. 14 days after the first male was introduced to the experimental female, vials were checked for the presence/absence of *bw* offspring, indicating whether the female remated or not. With the low offspring density of these vials, 14 days was sufficient time to ensure the vast majority of offspring reached adulthood before being counted. *D. melanogaster* shows strong last male sperm precedence [33], [34], so it is unlikely that we would incorrectly categorize a remated female as singly mated.

We carried out 100 trials for each experimental population (800 trials total). Trials were split evenly between two blocks, separated by one day. Because females had the opportunity to lay eggs in the same vial over the course of the entire assay (i.e. since her first mating), offspring from both sires should be present if the female remated. Vials in which wild-type offspring were absent, suggesting that the female did not mate with the first male, were discarded (71 trials). In addition to determining the presence or absence of any *bw* offspring, we also counted the number of *bw* and wild-type offspring for all females that remated (i.e. in which both types were present), providing an estimate of the relative number of offspring sired by each male. Variation in the relative number of offspring sired may arise from the time to each mating, sperm competition, and cryptic female choice. As females from each population were subjected to the same combination of stock then *bw* male, variance in remating and offspring production among female populations can be attributed to the effect of the female's population and/or the environment in which the population evolved. Because male identity and the order in which males were presented to females was held constant, we cannot detect any potential female × male interactions with respect to remating or paternity. However, we can use our measurements (and those outlined below) to detect overall population or environment-level differences in such sexual traits, as has been done previously in this species [35].

### Male reproductive defense

The second assay assessed whether males from the eight experimental populations varied in their influence on female remating and reproductive output. In each trial, a single stock female was placed together with a single experimental male (i.e. a male from one of the cadmium or ethanol-adapted populations) in a vial of standard food for 6 h during which the female could mate and lay eggs. The male was then immediately replaced with a *bw* male and the pair was left for another 8 h, at which point the second male was removed. Females were then allowed to lay eggs for 17 h, as above. 14 d after the beginning of the experiment, offspring were scored for eye color as a proxy for the occurrence of remating. If remating occurred, we again counted the number of wild-type and *bw* offspring to gain an additional measure of the first male's reproductive defense among remated females. This measurement is similar to that of sperm defense (P1) except that we also counted offspring produced between the first and second mating, thereby including variation among males in their time to mating. Trials were split evenly between two blocks, separated by one day. In this assay, the identity of the females and the competitor males were held constant for all trials such that variation in male remating defense and offspring production can be attributed to the experimental first male's population and/or environment. As above, 100 trials were performed for each male population (800 trials total), and data from females who did not mate with the first male were discarded (75 trials).

### Male reproductive offense

The third assay tested whether males from our experimental populations differed in their ability to induce mated females to remate, and their success at siring offspring of these females. In each trial, a stock female was first mated to a *bw* male and then given the opportunity to mate with a second male from one of the eight experimental populations. All trials were conducted within a single block. Offspring were scored to determine whether remating occurred (i.e. presence of wild-type offspring) and, for those that remated, the proportion of offspring sired by each male. This last measure is similar to sperm offense (P2) except that it includes offspring produced between the first and second matings. 110 trials were conducted for each male population (880 in total).

### Statistical analysis

For all three assays, variation in the propensity to remate was analyzed using a general linear mixed model in which the environment of the target individual was a fixed effect and population was a random effect nested within environment. Experimental block was also included as a fixed effect. The model was fit via restricted maximum likelihood (REML) via the mixed procedure in SAS v. 9.3 (SAS Institute, Inc., Cary, NC), with significance of the fixed effects determined using F-ratios and significance of the random effect of population determined using a likelihood ratio test (LRT). These analyses rely on the Gaussian approximation to the binomial distribution, which performs best when the probability of either outcome (i.e. remated or not) is approximately equal [36]. This was not the case in all our assays, so we repeated the above analyses employing a generalized linear model that specified a binomial distribution and a logistic link function, fit via maximum likelihood, as implemented in the Genmod procedure in SAS. Population was modeled as a fixed effect in this case and significance was determined via LRT's. Because results did not differ qualitatively between these two approaches, we present only the mixed model results.

In each assay, variation in the proportion of offspring sired by each male was tested using the same general linear mixed model with experimental block and environment as fixed effects and population as a random effect nested within environment. Results were qualitatively identical if proportions were arcsine square root transformed prior to analysis, so we present results of the non-transformed analyses.

## Results

### Female remating and offspring production

**Figure 1:**
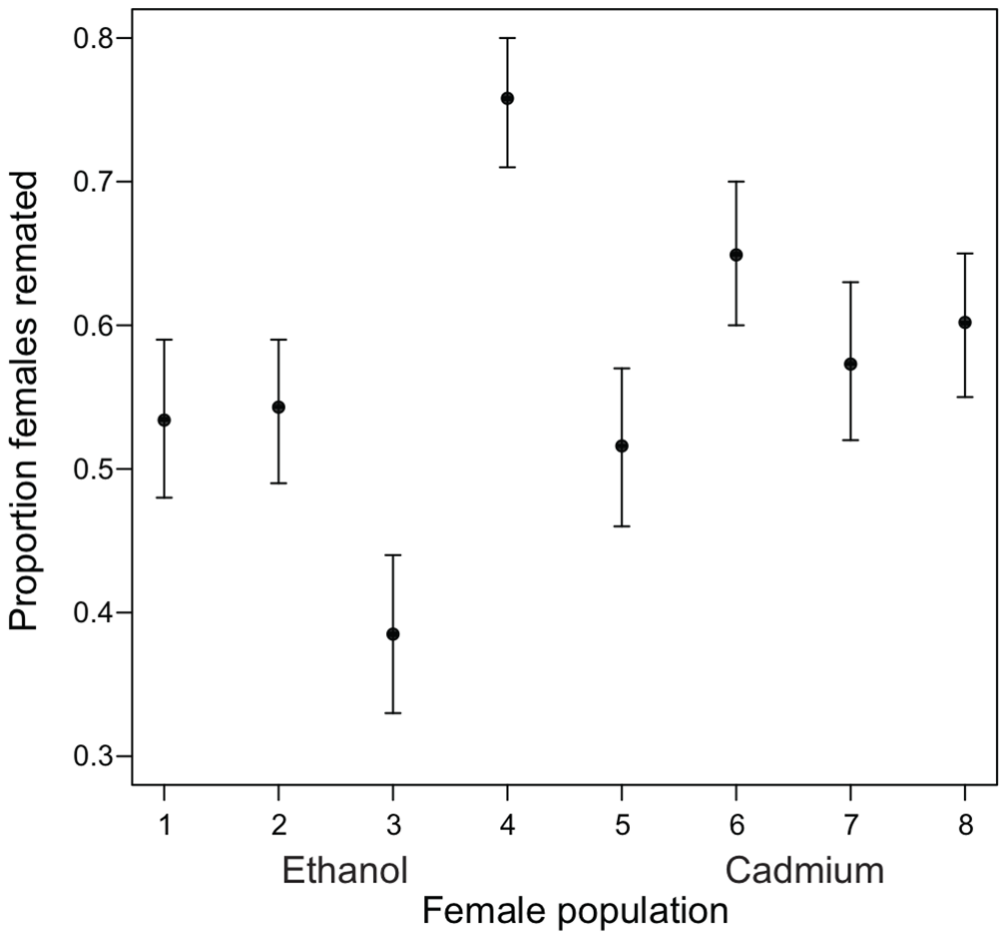
The proportion of experimental females that remated. Points are population-specific proportions (± SE) from the female remating assay. Populations 1–4 are ethanol-adapted, while populations 5–8 are cadmium-adapted.

**Figure 2:**
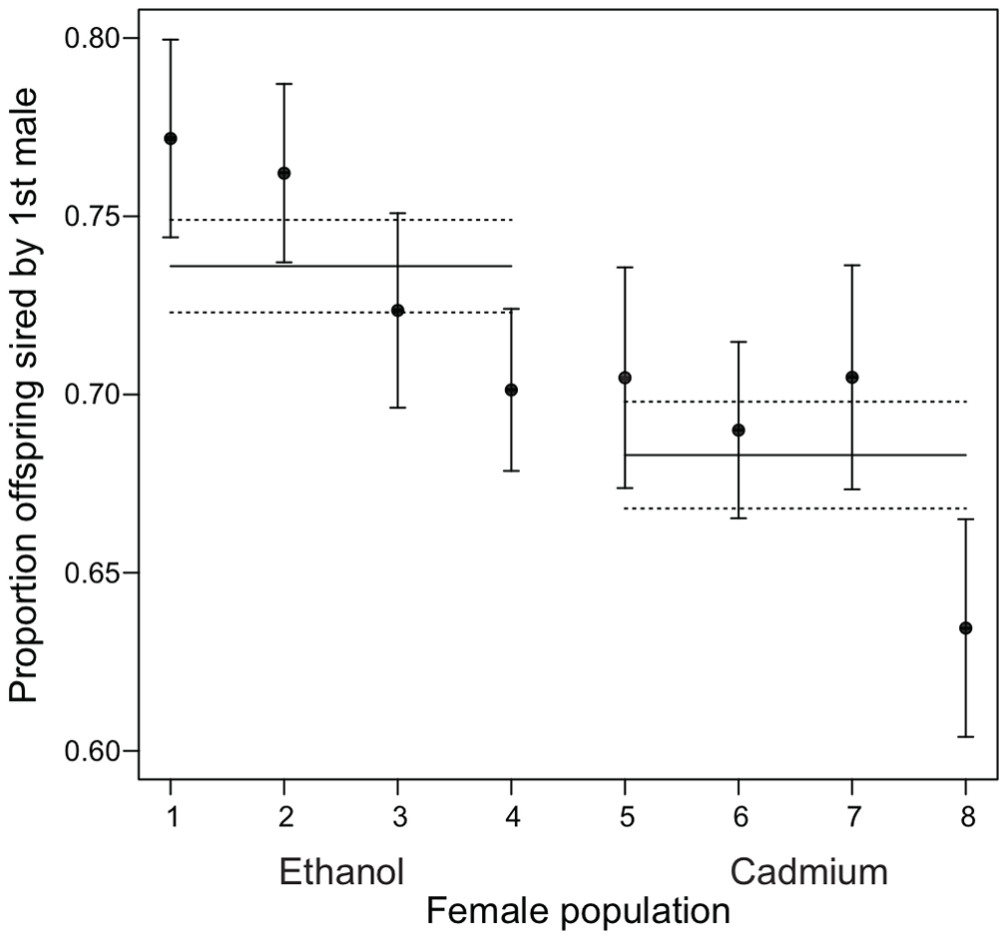
The proportion of offspring sired by first mates of experimental females that mated twice. Points represent population means (± SE). Horizontal lines represent the environment-level means for all ethanol and cadmium-adapted populations respectively (± SE, dashed lines). Populations 1–4 are ethanol-adapted, while populations 5–8 are cadmium-adapted.

There was significant among-population variation in the propensity of females from the eight experimental populations to remate (LRT: χ^2^_1_ = 15.2, p<0.0001; Fig. 1), indicating the evolutionary divergence of this trait among the eight populations. There was no evidence, however, that remating propensity differed on average between females adapted to the cadmium vs. ethanol environments (F_1,6_ = 0.13, p = 0.732). Among the females that did remate, among-population variation in the proportion of offspring sired by the first vs. second male was not significant (LRT: χ^2^_1_ = 0.78, p = 0.38). However, in females from the ethanol-adapted populations, a higher proportion of offspring tended to be sired by the first male than in females from the cadmium-adapted populations (Fig. 2). This difference between environments was marginally non-significant (F_1,6_ = 5.08, p = 0.065). There was no significant difference in the number of offspring produced by twice-mated females adapted to cadmium vs. ethanol (F_1,6_ = 0.09, p = 0.78), suggesting that the difference between environments in the proportion of offspring sired by the first male is not attributable to differences in overall egg laying rate.

### Male reproductive defense

There was no significant among-population variation in the ability of males to prevent stock females from remating (LRT: χ^2^_1_ = 0.12, p = 0.727), nor was there any evidence a male's ability to do this varied on average between populations adapted to the cadmium vs. ethanol environments (F_1,6_ = 0.39, p = 0.557; Fig. S1). Similarly, for those females that mated with both males, there was no significant among-population variation in the proportion of offspring sired by the first vs. second male (LRT: χ^2^_1_ = 0.73, p = 0.39), nor did this vary on average when the first males were adapted to the cadmium vs. ethanol environments (F_1,6_ = 0.89, p = 0.38; Fig. S1).

### Male reproductive offense

There was no significant among-population variation in the ability of a male to acquire a mating from a previously mated stock female (LRT: χ^2^_1_ = 0.004, p = 0.950), nor was there any evidence that this varied on average between males from populations adapted to cadmium vs. ethanol (F_1,6_ = 1.82, p = 0.226; Fig. S2). Likewise, for those females that mated with both males, there was no significant among-population variation in the proportion of offspring sired by the first vs. second male (LRT: χ^2^_1_ = 0.019, p = 0.896), nor did this vary on average between second males adapted to the cadmium vs. ethanol environments (F_1,6_ = 0.35, p = 0.547; Fig. S2).

We also found that the average remating rate among females in this assay was noticeably higher (87%) than that in the male reproductive defense assay (49%) in which the order of the same two types of competing males was reversed (Fig. S2). These assays were performed at different times (approximately one year apart) and this difference could therefore represent the effects of uncontrolled temporal variability. Alternatively, this difference could be due to the order in which the males were exposed to females, indicating that mutant males have a decreased ability to inhibit future matings in females they have mated and/or that experimental (wild-type) males are better able to overcome such inhibition in females. Similarly, siring success of first males was higher on average for the experimental males in the reproductive defense assay (70%) than those of the mutant males in the reproductive offense assay (52%). Again, this difference may be attributable to uncontrolled temporal variability, but it is possible that, relative to mutant males, the experimental (wild-type) males mated females faster than mutants and/or were better able to induce an increased egg-laying rate in these females.

## Discussion

Sexual conflict is suggested to be a potentially potent engine of diversity and speciation because it can drive the rapid evolutionary divergence of traits between allopatric populations even in the absence of ecological differences [2], [21], [22], [36]. Consistent with this, female propensity to remate differed significantly among our eight experimental populations, varying from as low as 38% to as high as 76% (Fig. 1). There was no evidence that this divergence was associated with adaptation to the different environments, with the most divergent populations both being adapted to ethanol. In contrast, when examining offspring production by these females, there was some evidence that the proportion sired by the first vs. second males differed between females adapted to the two environments. In particular, ethanol-adapted females tended to produce a higher proportion of offspring sired by the first male as compared to cadmium-adapted females, although this difference (means: 68% vs. 74% respectively; Fig. 2) was not quite significant (P = 0.065). Ecologically-dependent parallel evolution of traits involved in sexual conflict was shown previously in these populations with respect to male and female effects on female lifespan [32]. If borne out by further work, the extension of such effects to female reproductive traits involving male-male competition would further highlight a central role for ecology in the divergence of traits involved in conflict.

In contrast to these female reproductive traits, we found no evidence for the diversification of male traits involved in sexual conflict, either at the population or environment level. First, there was no significant among-population or between-environment variation in a male's ability to prevent stock females from remating, nor in their ability to acquire a mating from a previously mated stock female. Second, for those females that mated with both males, there was no significant among-population or between-environment variation in the proportion of offspring sired by the first vs. second male, independent of the mating order of the experimental male (i.e. first vs. second). The latter assay includes variation in the time to (re)mating, sperm competitive ability, and male-induced variation in female fecundity. Taken together, these results suggest that competitive male reproductive success, both defensive and offensive, did not diverge among the eight populations, nor between those adapted to ethanol vs. cadmium. However, we cannot exclude changes that are only manifest against coevolved females (and males) because we only examined males with stock females (and with *bw^D^* competitor males).

We previously found divergence among these eight populations in male harm that was associated with environment, wherein ethanol-adapted males reduced female longevity significantly more than cadmium-adapted males [32]. Ethanol-adapted females were also more resistant to this harm, on average, as compared to cadmium-adapted females. The lack of any environmentally-associated divergence in male reproductive defense and offense, as currently measured, implies that these traits do not underlie this previously observed ecologically-dependent parallel evolution of male harm. Long et al. [35] previously found significant differences in male remating defense, though not offense, among six replicate *D. melanogaster* populations independently adapted to the same environment, demonstrating divergence within a constant environment. It is unclear why no such divergence was detected here, although at more than 600 generations the Long et al. study had substantially more time for changes to accumulate. All of our populations were also evolving in novel environments in which directional selection was likely strong. Previous simulation results [32] and an empirical study [27] suggest that stronger natural selection may limit evolutionary exaggeration via sexual conflict, potentially explaining the lack of evolved differences in males. Finally, our assays also employed young flies and included only the first mating for the experimental males. It is therefore possible that effects could be detected in subsequent matings with older and more experienced flies. However, this design is similar to those that have been previously used and which have inferred ongoing sexual conflict [3], [35], [37].

As noted above, there was a trend in which ethanol-adapted females tended to produce a higher proportion of offspring sired by the first male as compared to cadmium-adapted females. If real, there are several potential explanations. First, ethanol-adapted females could take longer to remate, possibly because they have a longer refraction period between matings and/or because they more vigorously resist mating attempts by the second male, thereby giving them more time to lay eggs sired by the first male. More vigorous resistance could arise if the ethanol environment selects for more robust genotypes than does cadmium, a mechanism that could also explain why these females are more resistant to male-induced reductions in their longevity and why ethanol-adapted males are more harmful on average [32]. Alternatively, ethanol-adapted females could lay eggs faster after their initial mating, either because this is adaptive in ethanol or because they are differently affected by (i.e. resistant to) male seminal proteins [6], [38], [39]. Lastly, it is possible that females from different environments vary in their effects on sperm competition. Sperm competition is known to be influenced by male genotype [40], female genotype [41], and complex interactions between the rival males and females [42], [43]. Additional experiments would be required to determine whether the observed difference in proportion of offspring sired by the first male is primarily due to remating latency, egg-laying rate, or sperm competition (P2) effects. Regardless of the mechanism, our current results suggest that the traits involved in sexual conflict may often not evolve by sexually antagonistic selection alone, and that divergence via sexual conflict should not be considered an entirely non-ecological process.

Mating rate has large impacts on male and female fitness and is one of the classic examples of a trait under sexual conflict, wherein male fitness increases with increasing mating rate while female fitness is optimized at an intermediate rate [2], [44]. The fitness costs to females of mating beyond their optimum are well established in *D. melanogaster* [3], [5], [7], [37], [45] and remating rate (measured using similar methods as ours) has been shown to be negatively correlated with female resistance to male harm [37]. The fact that we and others [27], [36] have found divergence in this key component of sexual conflict within a constant environment provides strong evidence that sexual conflict can promote diversification in the absence of ecological differences, supporting its interpretation as a potential engine of speciation. However, given evidence that traits under sexual conflict may diverge in association with ecology [27], [30], [32], the interaction of within-environment diversification with ecologically-based divergent selection is an important topic for future work.

## Supporting Information

Figure S1 **Male reproductive defense.** a) the proportion (± SE) of stock females that remated when first mated to males from each of the eight experimental populations. b) the mean proportions (± SE) of offspring sired by the experimental males among females that first mated to an experimental male followed by a *bw* male.
(TIFF)

Figure S2 **Male reproductive offense.** a) the proportion (± SE) of stock females that remated when first mated to *bw* males and were subsequently exposed to males from one of the eight experimental populations. b) the mean proportions (± SE) of offspring sired by the experimental males among females that first mated to a *bw* male followed by an experimental male.
(TIFF)
