# Supplementary figures and images for: Remating and Sperm Competition in Replicate Populations of Drosophila melanogaster Adapted to Alternative Environments
Source: PLoS One. 2014 Feb 25;9(2):e90207. doi: 10.1371/journal.pone.0090207 (PMC3934985; doi:10.1371/journal.pone.0090207)

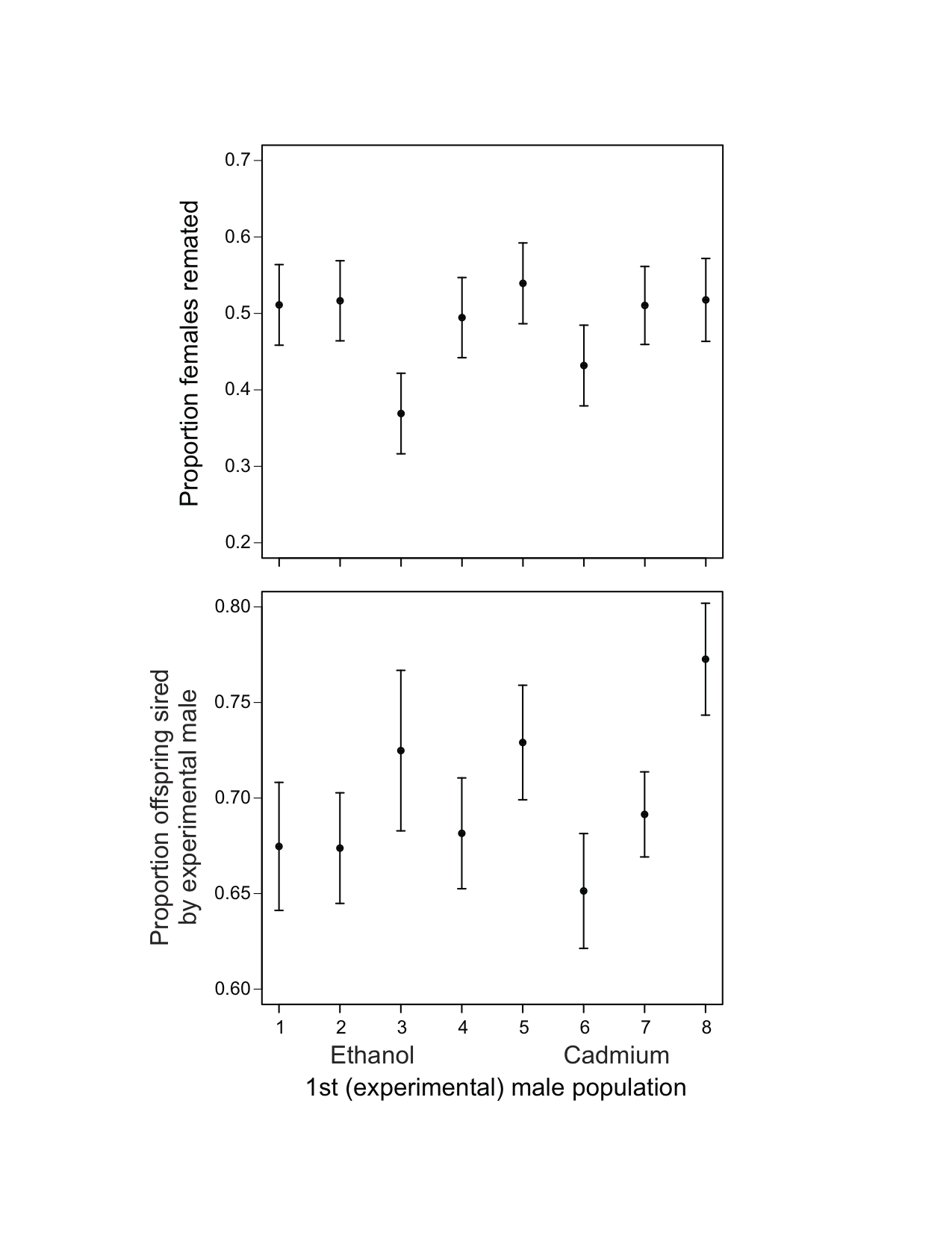

Supplement: Figure S1 — Male reproductive defense. a) the proportion (± SE) of stock females that remated when first mated to males from each of the eight experimental populations. b) the mean proportions (± SE) of offspring sired by the experimental males among females that first mated to an experimental male followed by a bw male. (TIFF) [file pone.0090207.s001.tiff]

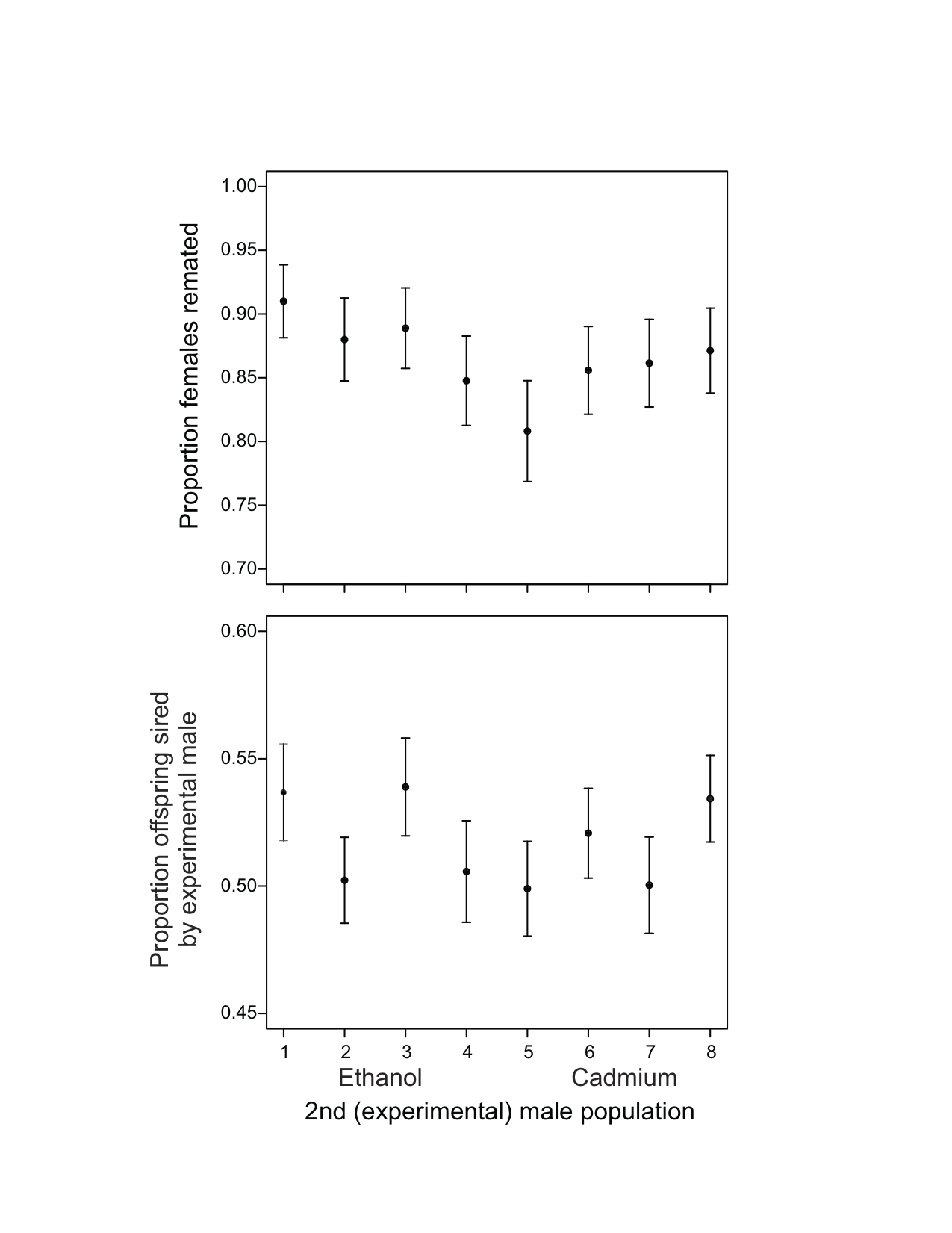

Supplement: Figure S2 — Male reproductive offense. a) the proportion (± SE) of stock females that remated when first mated to bw males and were subsequently exposed to males from one of the eight experimental populations. b) the mean proportions (± SE) of offspring sired by the experimental males among females that first mated to a bw male followed by an experimental male. (TIFF) [file pone.0090207.s002.tiff]
